# One-dimensional diffuse scattering of 1,3-di(*tert*-butyl)cyclopentadienyl pentaphosphaferrocene modelled with closed-form expressions

**DOI:** 10.1107/S2052520622007375

**Published:** 2022-08-18

**Authors:** Berthold Stöger, Eugenia Peresypkina, Alexander Virovets

**Affiliations:** a X-Ray Centre, TU Wien, Getreidemarkt 9, 1060 Vienna, Austria; b Institute of Inorganic Chemistry, Department of Chemistry and Pharmacy, University of Regensburg, Universitätsstraße 31, 93053 Regensburg, Germany; c Institute of Inorganic Chemistry, Goethe-University Frankfurt am Main, Max-von-Laue-Straße 7, 60438 Frankfurt am Main, Germany; University of Geneva, Switzerland

**Keywords:** polytypism, 1,3-di(*tert*-butyl)cyclopentadienyl pentaphosphaferrocene, disorder, diffuse scattering

## Abstract

1,3-di(*tert*-butyl)cyclopentadienyl pentaphosphaferrocene (Cp′′FeP_5_) crystallizes as polytypes, where adjacent layers are related by either a *c*- or an *n*-glide reflection. In the simplest possible polytypes only one kind of glide reflections is realized, leading to overall *Pca*2_1_ or *Pna*2_1_ symmetry respectively. Crystals isolated from the same crystallization experiment feature a varying degree of stacking disorder. One-dimensional diffuse scattering of a crystal with particularly strong disorder was modelled with closed-form expressions derived from a growth model. A range of interaction of *s* = 2 was necessary to satisfactorily describe the observed diffraction intensities. The crystal can be described as a disordered analogue of an allotwin, that is an intergrowth of the *Pca*2_1_ or *Pna*2_1_ polytypes with a distinct preference for the former.

## Abstract 2

The diffuse scattering of 1,3-di(*tert*-butyl)cyclopentadienyl pentaphosphaferrocene (Cp′′FeP_5_) is modelled with closed-form expressions derived from a growth model with the range of interactions (reichweite) *s* = 2.

## 1. Introduction

Disordered modular structures can be seen as a generalized form of crystalline matter where the arrangement of the individual (ordered) modules (layers, rods, bricks) is ambiguous and governed by probabilities. The case of layer structures with stacking disorder is well understood, since it can be described with simple growth models. In such models, the orientation of a layer depends on the orientation of a finite number *s* ≥ 1 [the *range of interaction* (Treacy *et al.*, 1991 ▸) or *reichweite* (Kakinoki & Komura, 1954 ▸)] of prior layers. In this context, the term ‘growth’ is to be understood formally with respect to the model, not the growth of a given crystal. The stacking arrangement of the crystal may also have been formed during a phase transition or by ageing.

The diffuse scattering caused by layer structures following a growth model has been intensely investigated [see *e.g.* Treacy *et al.* (1991 ▸) or Welberry (2010 ▸) and references therein]. Perhaps surprisingly, the function series used to calculate diffuse scattering are distinctly better behaved than the corresponding series of periodic structures, which typically do not converge at any point.

In particular, owing to pointwise exponential convergence, often only few layers have to be taken into account to adequately simulate diffuse scattering. In a sense, after these few layers, the ‘average’ structure, a weighted overlay of all the stacking possibilities, is obtained. This is the principle implemented in general programs such as *DIFFaX* (Treacy *et al.*, 1991 ▸).

However, in many common cases, instead of infinite series also closed-form expressions can be derived [see *e.g.* Jagodzinski (1949 ▸) and Kakinoki & Komura (1954 ▸)]. On the one hand, these are less general, as they are limited to special cases. On the other hand, they might be computationally more favourable and show no convergence problems in the highly correlated case. Moreover, they can be analytically differentiated for use in least-squares optimization problems. Above all, closed-form expressions may provide more insight into the shape of the diffraction peaks or the reason for homometry.

Recently, we described crystallization experiments of the ferrocene analogue Cp′′Fe(η^5^-P_5_), where one cyclopentadienyl (Cp, η^5^-C_5_H_5_^ −^) ring has been formally replaced by Cp′′ = η^5^-^*t*^Bu_2_C_5_H_3_^ −^, the 1,3-di-*tert*-butyl substituted analogue of Cp and the second Cp ring has been formally replaced by an aromatic η^5^-P_5_^ −^ ring. Different crystals extracted from the same sample featured different degrees of disorder (Peresypkina *et al.*, 2022 ▸). One of the crystals featured especially pronounced diffuse scattering with reflections indicating two different polytypes. Here, the diffuse scattering will be explained qualitatively and modelled quantitatively with growth model-derived closed-form expressions.

## 2. Experimental

### 2.1. Diffraction

Diffraction intensities from crystals of Cp′′FeP_5_ prepared according to Fleischmann *et al.* (2015 ▸) were collected at the P24 beamline of the PETRA III synchrotron, DESY (Germany) at 20 K (Peresypkina *et al.*, 2022 ▸). For all crystals, the structure was refined in the *Pca*2_1_ space group with more or less disorder of the Cp′′FeP_5_ molecule about the *y* = ¼ pseudo-reflection plane. For the crystal described below, the occupation of the two orientations was refined to 72.3:27.7 (3). The minor component was ignored for the modelling of the diffuse scattering. For further data collection and refinement details as well as atomic coordinates, see Peresypkina *et al.* (2022 ▸). Atomic form factors where approximated using the polynomial coefficients tabulated in Brown *et al.* (2006 ▸).

Reciprocal space sections were calculated using the *CrysAlisPro* software (Rigaku Oxford Diffraction, 2021 ▸). One-dimensional profiles were then extracted by summing up over 20 pixels perpendicular to the rod. The centres of the rods were determined visually.

## 3. Results and discussion

### 3.1. Stacking arrangements

**Figure 1:**
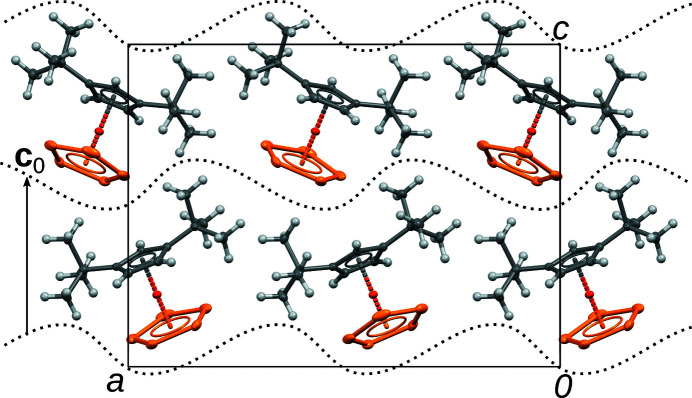
The *Pca*2_1_ structure of Cp′′FeP_5_ viewed down [010]. Fe (dark orange), P (bright orange) and C (grey) atoms are represented by ellipsoids drawn at the 50% probability level, H atoms by white spheres of arbitrary radius. Dotted curves mark the interface between layers.

The possible stacking arrangements have already been discussed in Peresypkina *et al.* (2022 ▸) and will be briefly recapitulated here. The structure is composed of ordered monoclinic/rectangular layers with *p*1*a*1 symmetry and one molecule located on the general position (Figs. 1 ▸ and 2 ▸). The translation lattice (henceforth simply lattice) of the layers is spanned by (**a**, **b**). The width of the layer is defined by the length of the **c**_0_ vector perpendicular to the layer plane.

**Figure 2:**
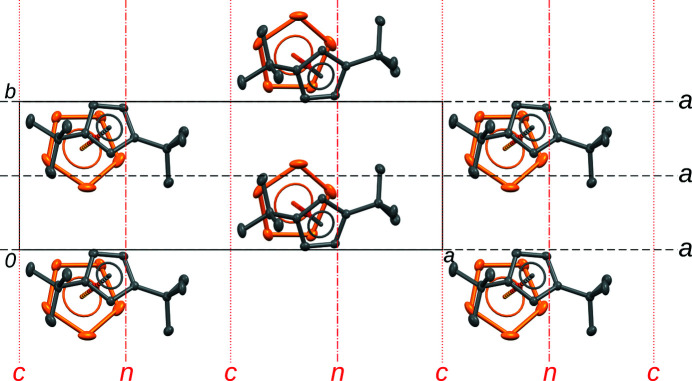
A single layer of Cp′′FeP_5_ molecules projected on the layer plane (001). The *a*-glide planes of the *p*1*a*1 layer group are indicated by the conventional symbols (Hahn & Aroyo, 2016 ▸) in black. Potential glide planes relating the layer to the next one are given in red. The symbols in this case are to be read with respect to the (**a**, **b**, 2**c**_0_) basis. H atoms are omitted for clarity.

Given the *n*th layer, the (*n* + 1)st layer can appear in one of two positions, which is generated from the *n*th layer by application of a glide reflections with the intrinsic translations **c**_0_ or **b**/2 + **c**_0_. These two operations will be called the *c*- and *n*-glide for convenience and are indicated using the corresponding graphical symbols in Fig. 2 ▸. The *c*-glide plane is located at *x* = 0 and the *n*-glide plane at *x* = ¼.

According to these rules, the origin of the *n*th layer can appear either at *n* **c**_0_ or at **a**/2 + **b**/2 + *n* **c**_0_. Thus, every polytype can be described by a family of integers 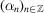 with α_*n*_ = 0, 1. The origin of the *n*th layer then is α_*n*_(**a** + **b**)/2 + *n* **c**_0_. If two adjacent layers *n* and *n* + 1 are related by a *c*-glide then α_*n*+1_ = α_*n*_, if they are related by a *n*-glide then α_*n*+1_ = 1 − α_*n*_.

### 3.2. MDO polytypes

In the context of OD theory, which describes polytypes that are locally equivalent, polytypes of a maximum degree of order (MDO) play a special role. The MDO polytypes of a family cannot be decomposed into fragments of simpler polytypes, *i.e.* polytypes of only a subset of *n*-tuples of adjacent layers (Dornberger-Schiff, 1982 ▸). MDO polytypes can be considered as the ‘alphabet’ of an OD polytype family: all polytypes can be decomposed into fragments of MDO polytypes. Moreover, in the majority of cases, ordered polytypes belong to the MDO class.

**Figure 3:**
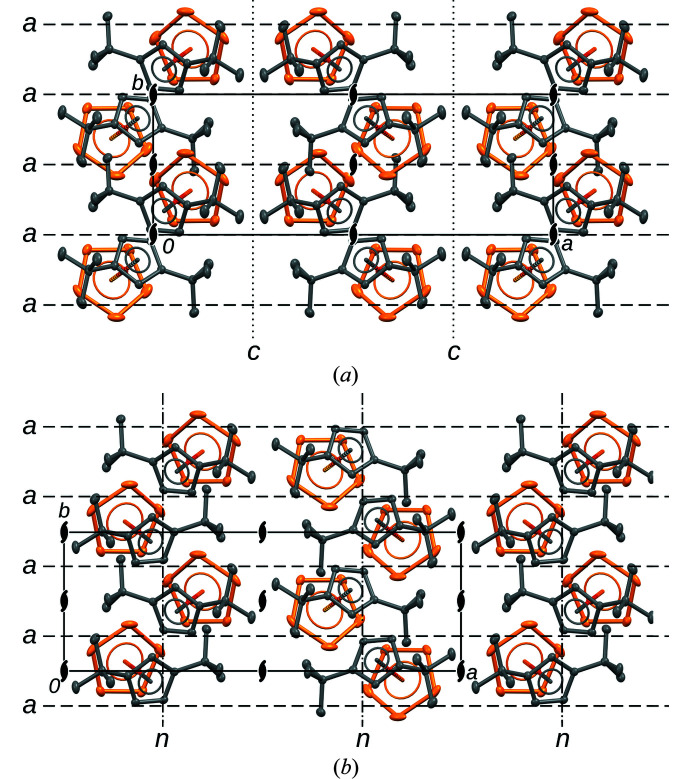
The (*a*) *Pca*2_1_ and (*b*) *Pna*2_1_ MDO polytypes of Cp′′FeP_5_ projected on the layer plane (001). Symmetry elements are indicated by the conventional symbols (Hahn & Aroyo, 2016 ▸). H atoms are omitted for clarity.

Cp′′FeP_5_ forms non-OD polytypes because pairs of adjacent layers related by *c*- or *n*-glides are not equivalent. However, the MDO concept is just as useful in this case. Here, there are two MDO polytypes: *Pca*2_1_ [all adjacent layers related by *c*-glides, (α_*n*_) = …, 0, 0, 0, 0, …] and *Pna*2_1_ [all adjacent layers related by *n*-glides, (α_*n*_) = …, 0, 1, 0, 1, …]. In both cases **c** = 2**c**_0_. The two MDO polytypes are shown in Fig. 3 ▸. Note that the 2_1_ screw rotations are at different positions with respect to the layers.

### 3.3. Qualitative interpretation of the diffraction pattern

**Figure 4:**
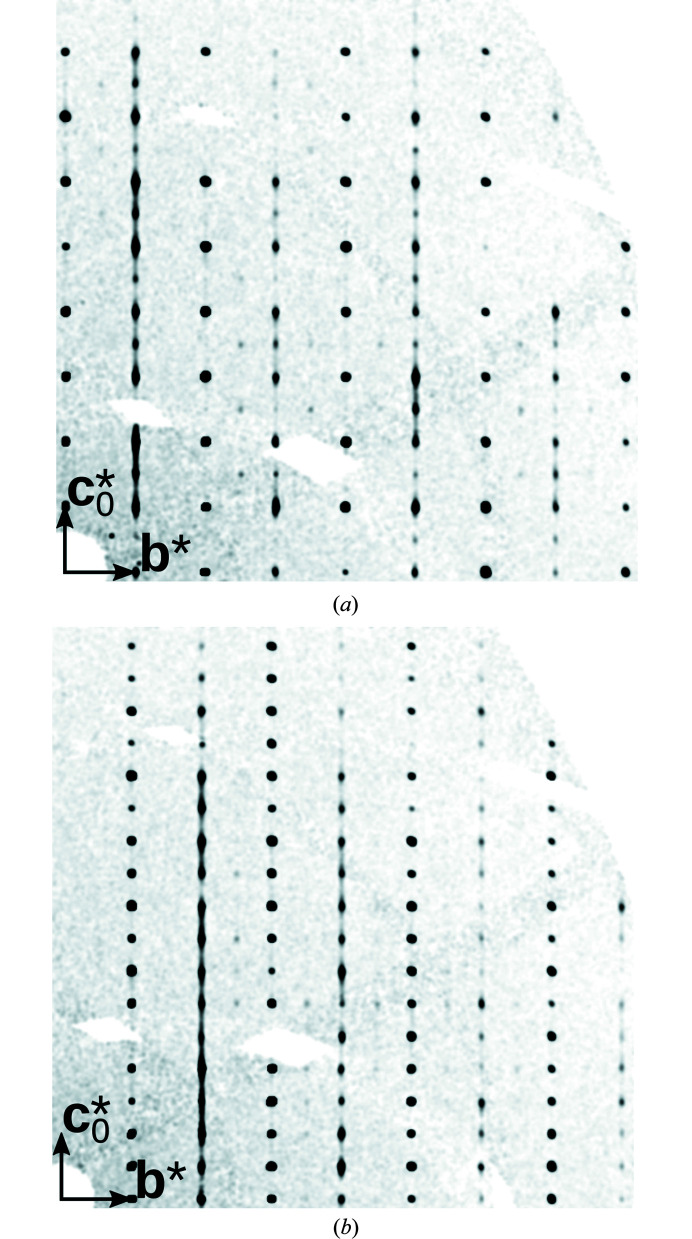
Sections through reciprocal space of a disordered Cp′′FeP_5_ crystal with (*a*) *h* = 0 and (*b*) *h* = 1.

Fig. 4 ▸ shows the diffraction pattern of the crystal under investigation. On rods *h* + *k* even only sharp reflections are observed, whereas on rods *h* + *k* odd, distinct diffuse scattering is apparent. The positions of all maxima are in agreement with a lattice spanned by (**a**, **b**, 2**c**_0_), which corresponds to either of the two MDO polytypes. Qualitatively, the polytypes can be differentiated by the systematic absences of the *c*_[100]_ or *n*_[100]_ glide reflections, respectively. For 0*kl* reflections where *k* is odd (for *k* even, both glide reflections feature the same reflection conditions), strong reflections *l* odd suggest the *Pca*2_1_ polytype. Additional weak reflections *l* even prove a non-negligible contribution of *Pna*2_1_ fragments [Fig. 4 ▸(*a*)]. Thus, it appears that the crystal is built of both polytypes. An intergrowth of two or more distinct polytypes is called an allotwin (Nespolo *et al.*, 1999 ▸). However, in contrast to the crystal described here, the domains of an allotwin are macroscopic.

Additional extremely weak reflections are observed at half-integral *k* values, which are due to superstructure formation in the [010] direction for the *Pca*2_1_ polytype as described in Peresypkina *et al.* (2022 ▸). These reflections are significantly more pronounced for pure *Pca*2_1_ crystals. But even there the modulation is minute. For the disordered crystal described here, these reflections can be neglected without hesitation.

### 3.4. Quantitative interpretation of the diffraction pattern

In the following, positions in reciprocal space will be expressed with respect to the reciprocal basis 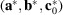 = 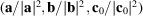 . The coordinates are given as *hk*ν to emphasize that diffraction intensities can only appear at integral *h* and *k* owing to the layer lattices, but diffuse scattering may appear along rods parallel to 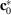 at arbitrary real ν values, in the case of disordered layer arrangements.

Let *F*_*n*_(*hk*ν), 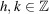 , 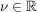 be the structure factor of the *n*th layer. Layers *n* even are translationally equivalent and therefore their structure factor only differs in phase from *F*_0_(*hk*ν). In contrast, layers *n* odd are reflected at [100] and thus will be expressed with respect to 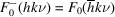 . For brevity, henceforth the argument *hk*ν of *F*_0_ and 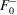 will be omitted. The structure factor of the *n*th layer is given by: 

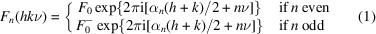

The overall diffraction intensity of a structure can be written as

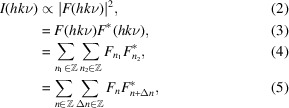

where an asterisk indicates the complex conjugate and Δ*n* designates the distance between layers. By separating into even and odd Δ*n* and substituting equation (1):

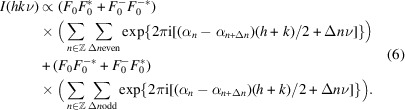

For *h* + *k* even, (α_*n*_ − α_*n*+1_)(*h* + *k*)/2 is integral and therefore *I*(*hk*ν) is independent of (α_*n*_). Thus, all polytypes produce the same intensity distribution on rods *h* + *k* even. In particular, by application of the Dirichlet kernel, one can show that intensities appear only for integral and half-integral ν: 

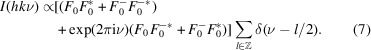

The term 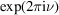 is 1 and −1 for integral and half-integral ν, respectively. These reflections are called *family reflections*, since they are identical for all members of the polytype family.

For *h* + *k* odd, the intensities differ according to the polytype and reflections on these rods are therefore called *characteristic reflections*. Let *P*_Δ*n*_ be the probability that α_*n*_ = α_*n*+Δ*n*_. For the MDO polytypes *P*_Δ*n*_ = 1 (*Pca*2_1_) and *P*_Δ*n*_ = [(−1)^Δ*n*^ + 1]/2 = …0, 1, 0, 1… (*Pna*2_1_), respectively. For α_*n*_ = α_*n*+Δ*n*_ the term (α_*n*_ − α_*n*+Δ*n*_)(*h* + *k*)/2 is 0, whereas for α_*n*_ = α_*n*+Δ*n*_ it is half-integer. Equation (6) thus becomes: 

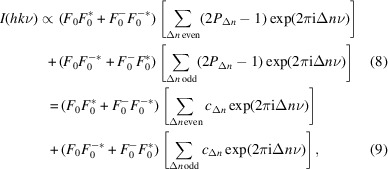

where 2*P*_Δ*n*_ − 1 is written as *c*_Δ*n*_, the correlation of the variables α_*n*_ and α_*n*+Δ*n*_.

### 3.5. The special case of the *h* = 0 plane

Qualitatively, the diffuse scattering is best understood for the *h* = 0 plane. As noted above, the *Pca*2_1_ and *Pna*2_1_ MDO polytypes can be immediately distinguished by reflections on the *k* odd rods. The former features sharp reflections at integral, the latter at half-integral ν values.

Quantitatively, the situation is likewise simpler, because *F*(0*k*ν) = *F* ^−^(0*k*ν) and equation (9) simplifies to 

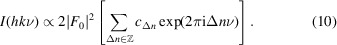

In the simplest growth model, the probability of a *c*-glide relating adjacent layers is given by *P* (and conversely the probability of an *n*-glide by 1 − *P*). However, such a simple model cannot explain the existence of both, the *Pca*2_1_ and *Pna*2_1_ reflections. In fact, these models produce on rods *h* + *k* odd streaks of the form 

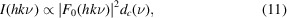

where *c* = 2*P* − 1 and *d*_*c*_ stands for the family of functions 

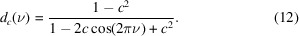

**Figure 5:**
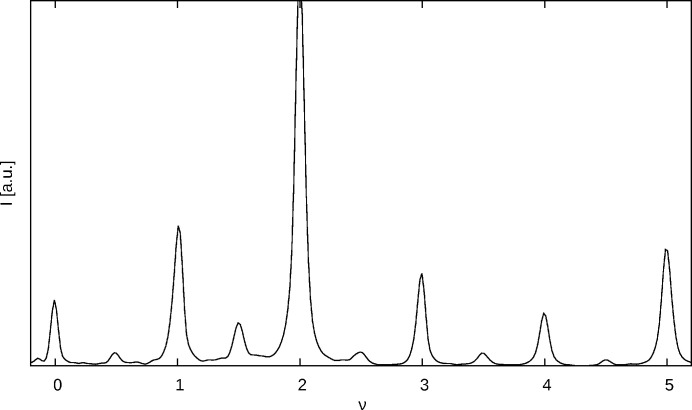
(01ν)* rod showing distinct peaks at integer and half-integer ν.

These functions are well known and generally describe the shape of nearest-neighbour (*s* = 1) models with the nearest-neighbour correlation *c* (Welberry, 2010 ▸; Stöger *et al.*, 2021 ▸). For *c* approaching 1 and −1, *d*_*c*_(ν) converges to a Dirac comb at integral and half-integral ν, respectively. With decreasing |*c*|, the peaks become successively broader and at *c* = 0, *d*_*c*_(ν) is constant. However, according to this model, in no case peaks are observed simultaneously for integral and half-integral ν, which is in contradiction with the observed intensities (Fig. 5 ▸).

Thus, a range of interaction *s* ≥ 2 is required. In an *s* = 2 two-neighbour growth model, the state of the previous step is considered as in the Markov chain: 
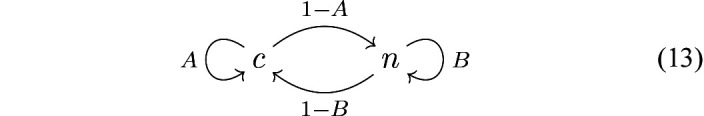


The parameter *A* gives the probability that a *c*-glide follows a *c*-glide and *B* that an *n*-glide follows an *n*-glide. For example, *A* = 1, *B* < 1 and *A* < 1, *B* = 1 represent the *Pca*2_1_ and *Pna*2_1_ polytypes, respectively and *A* = *B* = 0 is the ordered non-MDO polytype …*cncn*…. For *A* = 1 − *B*, the model degenerates to the single-neighbour model *P* = *A* = 1 − *B* described above.

Henceforth, degenerate Markov chains *A* = 1 or *B* = 1 as well as the ordered *A* = *B* = 0 model will be disregarded. Then, it is easy to show that the chain converges to an equilibrium state where the probability of *c*- and *n*-glides is 

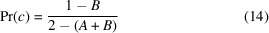

and 

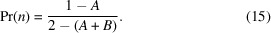

To calculate the diffuse scattering of a given model, the probabilities 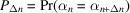 have to be calculated [see equation (9)]. Since α_*n*_ can adopt two values, the number of states is doubled to four: 
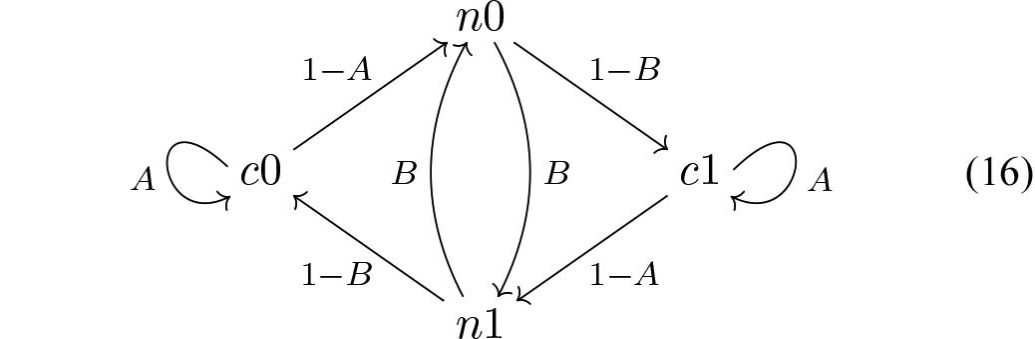


The state *c0* means a *c*-glide after α_*n*_ = 0, *etc*. The *n*th state of the Markov chain will be described by the row-vector 

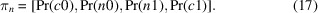

According to equations (14) and (15), the initial state of the chain is 

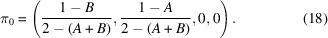

The transition probability matrix 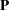 , which gives the progression of the chain according to 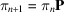 is 

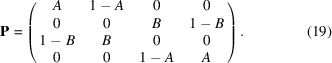

Therefrom, the diffuse scattering on rods *h* + *k* odd can be calculated as

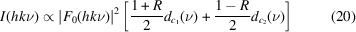

where 

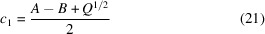




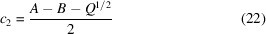




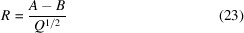




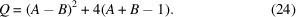

For derivation see Appendices *A*1 and *A*2 and also Kakinoki & Komura (1954 ▸).

**Figure 6:**
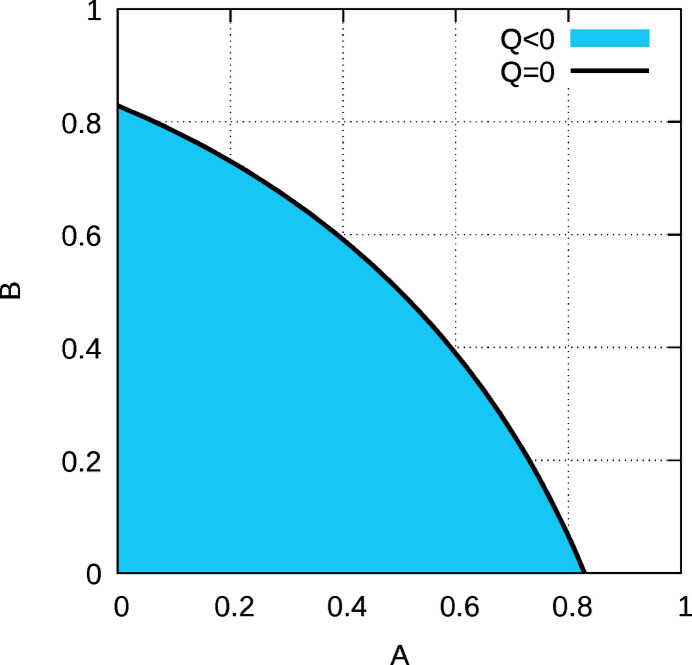
Sign of *Q* depending on *A* and *B*: white ’+’, blue ‘−’ and black 0.

**Figure 7:**
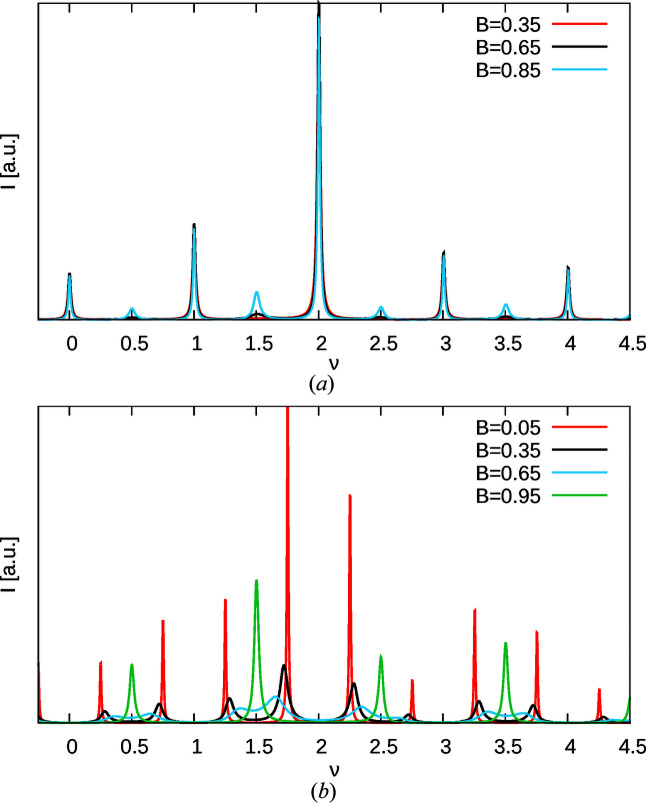
Simulation of the (01ν)* rod for (*a*) *A* = 0.95 and (*b*) *A* = 0.05 and selected values of *B*.

Two cases can be distinguished. For large *A* or *B* (white region in Fig. 6 ▸), *Q* is positive and (*Q*)^1/2^ is real. Equation (20) can then be understood as a linear combination of the diffuse scattering of two independent nearest-neighbour models with the correlations *c*_1_ and *c*_2_. Although the values of the two correlations and the weighting of the two terms is not immediately obvious, a qualitative analysis confirms the expectations: for large *A* and small *B*, sharp diffraction spots are observed at integral ν, corresponding to the *Pca*2_1_ MDO polytype [red curve in Fig. 7 ▸(*a*)]. With increasing *B*, additional reflections slowly appear at half-integral ν, corresponding to the *Pna*2_1_ polytype (black and blue curve). Ultimately, for *A* = *B* the diffraction pattern corresponds to that of a mix of *Pca*2_1_ and *Pna*2_1_ polytypes with equal volume weights and peaks enlarged along c*.

With small *A* and *B* (blue region in Fig. 6 ▸), *Q* is negative and therefore (*Q*)^1/2^ and *R* are purely imaginary. The case is shown in Fig. 7 ▸(*b*) for small *A*. For large *B* ‘reflections’ of the *Pna*2_1_ polytype are observed (green curve). With decreasing *B*, these reflections split (black and blue curve). Ultimately, when *B* approaches *A* (red curve), reflections are observed at uneven fourths of ν, which corresponds to the …*cncn*… non-MDO polytype with **c** = 4**c**_0_, leading to the doubling of the cell parameter *c* with respect to the experimentally observed polytypes. Reflections at integral and half-integral values are not observed, because this particular polytype features *Pna*2_1_ symmetry.

The problem of the imaginary value of (*Q*)^1/2^ in the case of *Q* < 0 can by solved by a purely real form of equation (23), given in Appendix *A*2. It might be more appropriate for calculations, but since the diffraction pattern from the crystal does not suggest this case, it is not discussed any further.

For *Q* approaching 0 (black line in Fig. 6 ▸), the denominator of *R* goes to 0 and *R* diverges. However, in that case *c*_1_ = *c*_2_ = *c* = (*A* − *B*)/2 and *R* can be set to an arbitrary value (such as *R* = 0) to get 

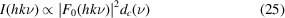

which is the diffraction pattern of a nearest-neighbour model with correlation *c* = (*A* − *B*)/2 [see equation (11)].

### 3.6. Interesting special cases: homometry

**Figure 8:**
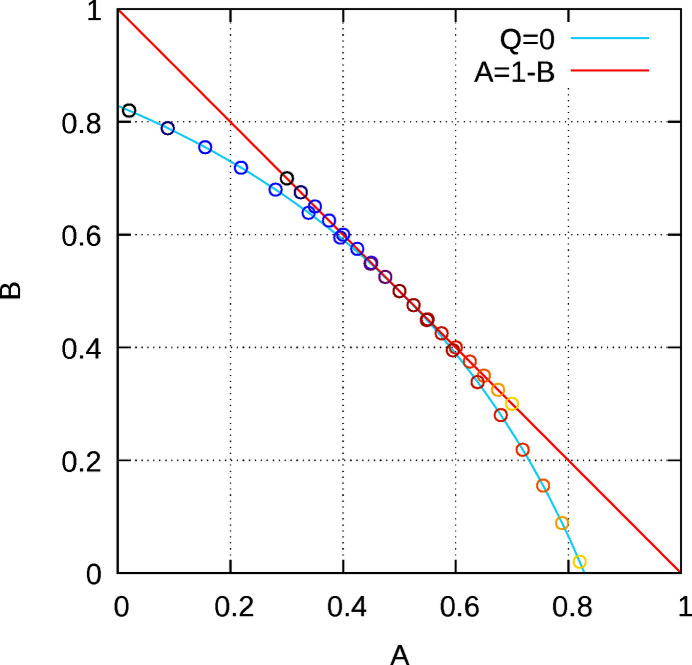
Parameters *A* and *B* of models with a diffraction pattern corresponding to a nearest-neighbour model. Red line: actual nearest-neighbour model (*A* = 1 − *B*); blue line: *Q* = 0 [*B* = *A* + 2(2)^1/2^(1 − *A*)^1/2^ − 2]. Pairs of circles with the same colours indicate example pairs of homometric structures.

The case *Q* = 0 is particularly interesting, because it shows that diffuse scattering can be inherently homometric, which means that different structures produce identical diffraction patterns. Indeed, for |*c*| < (2)^1/2^ − 1 the two-neighbour growth model *A* = 1 − (1 − *c* ^2^)/2 and *B* = 1 − (1 + *c*)^2^/2 (with *Q* = 0), produces the same diffraction pattern as the nearest-neighbour model *A*′ = (1 + *c*)/2 and *B* = 1 − *A* = (1 − *c*)/2. However, these parameters correspond to *different* stacking arrangements. Fig. 8 ▸ shows such pairs of parameters of homometric structures.

This kind of homometry is well known (Welberry, 2010 ▸) and has to be taken into account when determining the parameters of the growth model. In fact, homometry or quasi-homometry may lead to local minima and therefore a local search may not be sufficient.

### 3.7. Estimation of the parameters *A* and *B* from a *h* = 0 rod

As noted above, the crystal under investigation appears to contain the *Pca*2_1_ and *Pna*2_1_ MDO polytypes with a distinct preference for the former. Thus, quantitatively one would expect probabilities *A* > *B* > ½.

To derive *A* and *B*, a rod *h* = 0, *k* odd was chosen where the diffraction intensities were strong and the peak maxima were consistent with the integrated diffraction intensities. The (01ν)* rod was the optimal rod in that regard.

The high-quality diffraction data obtained with the high-flux non-divergent synchrotron beam and a noiseless direct photon-counting detector at the P24 beamline (DESY, Hamburg) operated in shutterless mode allowed very narrow scans (0.1° 2θ). Thus, little experimental broadening is expected, which makes it possible to reliably reconstruct and quantify the diffuse scattering.

**Figure 9:**
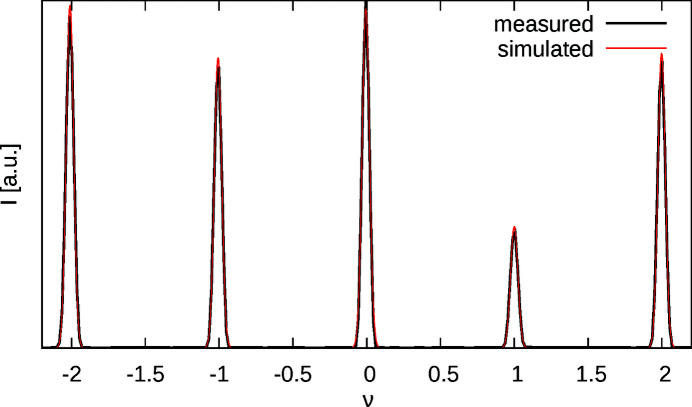
Measured intensities of the (02ν)* rod and calculated profile using Gauss functions with σ = 2.96646 pixels.

The experimental peak broadening was approximated by a Gaussian distribution, whose variance σ^2^ was determined by least squares refinement against the sharp family reflections (02*l*) (Fig. 9 ▸).

Intensities of the (01ν)* rod were calculated using the analytical expression equation (20) and numerically convoluted with the Gaussian peak broadening. The scale factor was determined by linear regression after each calculation.

**Figure 10:**
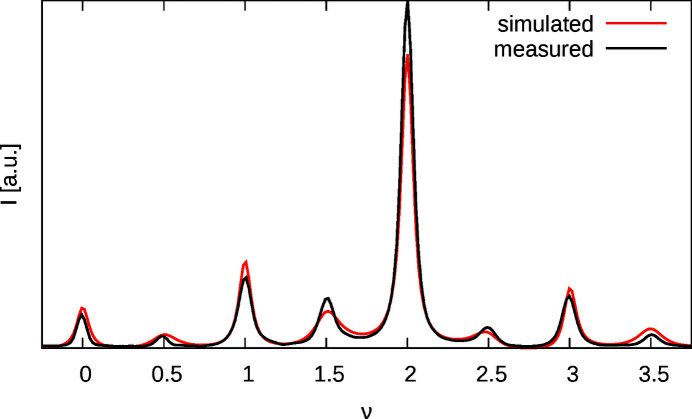
Measured and simulated intensities of the (01ν)* rod.

The origin (ν = 0) and the length | 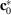 | in pixels as well as *A* and *B* were determined using a combination of global search with local searches according to the multi-coordinate search (MCS) approach (Huyer & Neunmaier, 1999 ▸) (Fig. 10 ▸) implemented in custom routines. The minimized function was the square of the difference between measured and calculated intensities with unit weight:

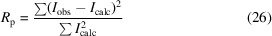

Since each rod is calculated in ms times on a standard computing hardware, the search ended in a short time with a final residual of *R*_p_ = 1.2 %. Only one ‘basket entry’ (Huyer & Neunmaier, 1999 ▸) was obtained, which means that the function had only a single local minimum. Thus, homometry was not a problem in this case.

The refined values are summarized in Table 1 ▸, left row. As predicted, *c*-glides induce *c*-glides (83.1% probability) and *n*-glides induce *n*-glides (61.1% probability). In that sense, as we had expected, the crystal can be considered as a disordered equivalent of an allotwin. In contrast to the crystal under investigation, though, an allotwin is composed of macroscopic domains, *i.e.* *A* and *B* are very close to 1.

Since the *c*-glide induces the *n*-glide with lower probability than the *n*-glide induces the *c*-glide (16.9% versus 38.9%), the *Pca*2_1_ fragments prevail in agreement with the experimentally observed systematic absences and relative weight of the disordered components in structure refinements.

### 3.8. The general case of planes *h* ≠ 0

The *h* ≠ 0 planes are more difficult to interpret quantitatively, because the characteristic reflections produced by both MDO polytypes overlap. The simplification *F*(0*k*ν) = *F* ^−^(0*k*ν) does not apply and the general equation (9) has to be used. The intensity distribution for rods *h* + *k* odd then calculates as

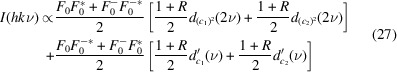

where *c*_1_, *c*_2_ and *R* are defined as in the *h* = 0 case and *d*′_*c*_ represents the family of shape functions 

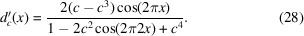

For derivation see Appendix *A* [App appa]3.

The shape functions of the first term are weighted by the average of the auto-correlations 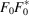 and 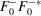 . Note that here, the correlations *c*_1_ and *c*_2_ enter as their squares and the argument of the shape function *d* is 2ν instead of ν. This is reasonable, because the term considers only layer pairs distanced by an even number Δ*n* of layers.

The shape functions of the second term are weighted by the average cross-correlations 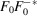 and 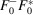 . This average is real, but may be negative. However, the sum of auto- and cross-correlations is 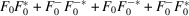 = 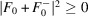 , which ultimately prevents negative intensities. Owing to the two different terms in equation (27), the peaks can adopt a distinctly skewed shape.

**Figure 11:**
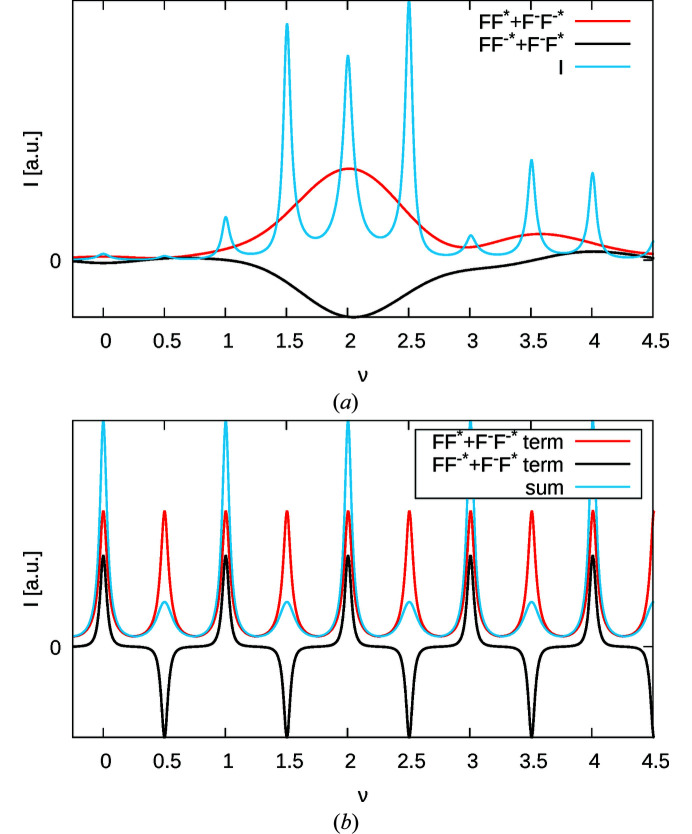
Calculated (21ν)* rod for *A* = 0.85, *B* = 0.75. (*a*) Overall intensity *I*(21ν) and the 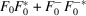 and 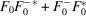 factors. (*b*) The 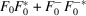 and 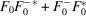 terms of equation (27) if *F*_0_ and 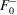 were constant and equal. The sum of the two terms is also shown.

To exemplify equation (27), Fig. 11 ▸(*a*) gives the sum of auto-correlations 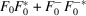 , the sum of the cross-correlations 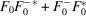 and the intensity of a (21ν)* rod for the parameters *A* = 0.85 and *B* = 0.75.

Fig. 11 ▸(*b*) plots the profiles of the reflections of equation (27) under the assumption of constant and equal *F*_0_ and 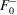 and the dissection into the two contributing terms.

**Figure 12:**
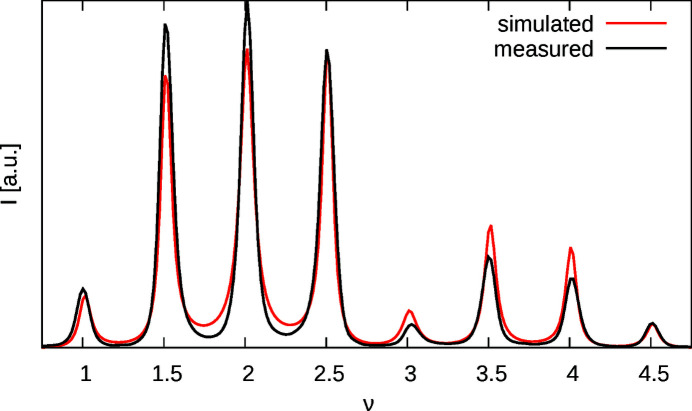
Measured and simulated intensities of the (21ν)* rod.

The result of a MCS refinement using equation (27) is shown in Fig. 12 ▸ and summarized in Table 1 ▸. The fit is still reasonable, though slightly worse than in the (01ν)* case.

### 3.9. Significance of refinements

Comparing the refinements on the (01ν)* and (21ν)* rods, the *A* values agree well. However, a distinctly higher *B* value was derived from the (21ν)* rod. Indeed, since the contribution corresponding to the *Pna*2_1_ MDO polytype is distinctly less pronounced (*R* > 0), the *B* parameter is defined worse than the *A* parameter. This can be seen by plotting the loss function against the *A* and *B* parameters with fixed metric parameters. The minimum lies in a valley that is much steeper in *A* than in *B* direction.

**Figure 13:**
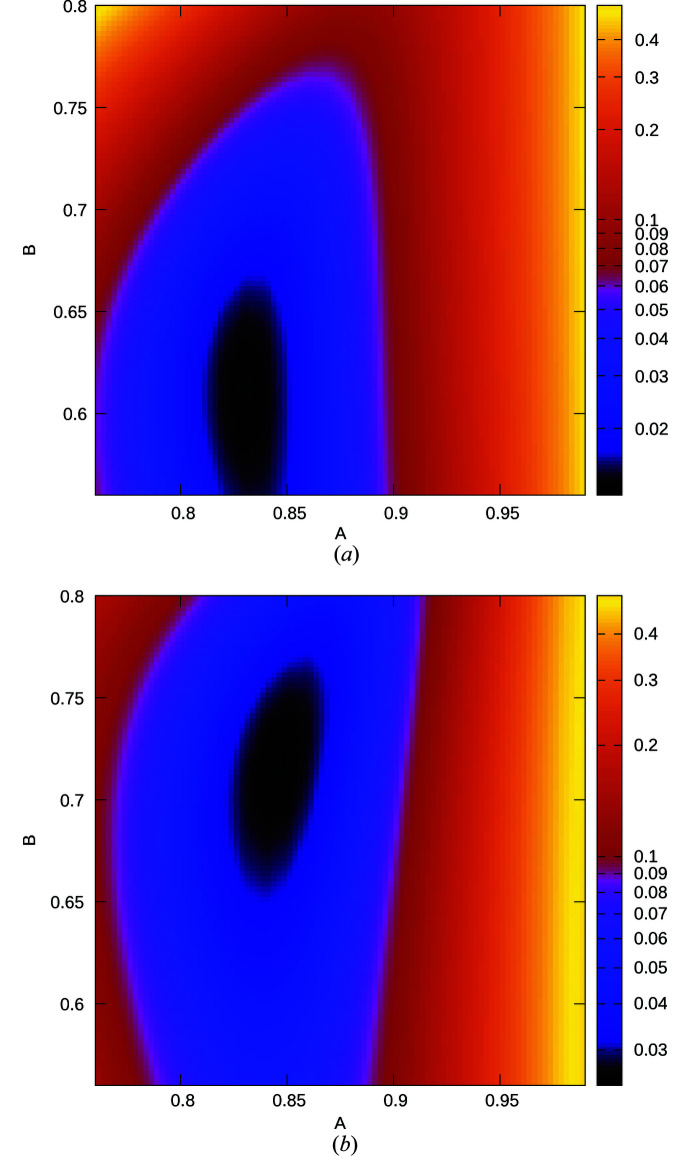
(01ν)* (21ν)* *R*_p_ values depending on *A* and *B* for the (*a*) (01ν)* and (*b*) (21ν)* rods with the final metric parameters (centre and |**c**_0_| in pixels) as given in Table 1 ▸. *R*_p_ values are encoded by colours on a logarithmic scale.

The difference in the *B* value cannot however be explained by imprecision alone, since the valleys are located at distinctly different positions (Fig. 13 ▸), systematically shifted toward the larger *B* values for the (21ν)* rod.

The stability of *A* and the higher *B* for *h* ≠ 0 is confirmed by additional refinements against the (03ν)* an (23ν)* rods (Table 2 ▸). These refinements are less reliable, because the intensities on these rods are significantly weaker and the contribution of *B* is even less significant.

For *k* = 5 rods, the extracted intensities are too inconsistent with the structure factors for reasonable refinements. Likewise for *h* = 1 rods with even *k* contribution of *B* to the shape was too small for a meaningful determination of this value.

One source of error might be inadequate intensity extraction. Indeed, the relative peak heights sometimes disagree with the integrated *I*_*hkl*_ values. However, we suppose that the biggest error is due to neglecting desymmetrization. A layer can appear in three unique environments with respect to the two neighbours: *cc*, *nn* and *cn* (= *nc*). However, structural data only exists for the *cc* (corresponding to the *Pca*2_1_ MDO polytype) case. For improved simulations, one would either have to grow crystals featuring the other layer contacts or resort to theoretical structure optimizations.

For the (01ν)* rod this error seems to be minimized. In fact, the *FF* ^−*^ + F*F ^−^ cross-correlation term does not appear in the analytical expressions, since in projection along [100] both orientations are identical. We suppose that also the desymmetrization is less pronounced in the [100] projection, leading to a more reliable estimation of *B*. Moreover, in that case, the values of *A* and *B* are derived from distinct ‘reflections’, whereas for *h* ≠ 0, they are ‘encoded’ in the same reflections.

## 4. Conclusion and outlook

Having high-quality diffraction data for single crystals of Cp′′FeP_5_ obtained with high-flux synchrotron radiation at the P24 beamline (DESY, Hamburg), we attempted to describe the shape of the experimentally observed one-dimensional diffuse scattering with closed-form expression derived from a growth model with range of interaction *s* = 2 and explain the average disorder in the structure (Peresypkina *et al.*, 2022 ▸). Thus, a direct relation between the stacking-fault probabilities of both MDO polytypes and the form of the diffraction maxima could be drawn.

Closed-form expressions allow for very fast calculations of diffuse scattering, which we used for respective refinements. The fact that tens of thousands of rods can be calculated in seconds enabled a global search. This is crucial in the case of diffuse scattering, since homometry may lead to multiple local minima. Moreover, closed-form expressions directly explain the shape and position of peaks in the diffuse scattering and allow a direct derivation of homometric pairs of disordered stacking arrangements.

However, such an approach is not general. Currently, deriving the expressions for diffuse scattering intensity is tedious and error-prone. In the future this might be automatized by symbolic algebra, since the theory of Markov chains is well understood. Even for the title compound, owing to the different orientations of the even and odd numbered layers, the general expressions are rather unwieldy. These complications disappeared, when only considering the (0*k*ν)* plane. Effectively, this means looking at a projection along [100], for which the orientation with respect to [100] vanishes. Then, the diffuse scattering can be described as the sum of two nearest-neighbour models. In the general case (*h* ≠ 0), however, the expressions become less intuitive. With an increasing range of interaction or number of orientations, the mathematical expressions will become more and more cumbersome. A fundamental complexity limit is due to tha fact that, in general, only roots of polynomials up to degree four can be expressed by radicals. Thus, for more complex problems, only numerical solutions can be given.

The imperfect fit is most likely due to ignoring desymmetrization effects. We will show such a case in an upcoming publication of a different molecule, where two kinds of polytypes could be grown and thus desymmetrization could be taken into account. When considering different environments, Markov chains with yet more states have to be used. 

## Figures and Tables

**Table 1:** Results of the simulation of the (01ν)* and (21ν)* rods *c*_1_, *c*_2_, *R*, Pr(*c*) and Pr(*n*) were derived from *A* and *B* and not explicitly refined.

Parameter	(01ν)*	(21ν)*
*R*_p_	0.012	0.025
*A*	0.831	0.848
*B*	0.611	0.721
*c*_1_	0.784	0.820
*c*_2_	−0.564	−0.693
*R*	0.421	0.305
Pr(*c*)	0.697	0.647
Pr(*n*)	0.303	0.352
Centre (pixels)	521.39	520.56
|**c**_0_| (pixels)	29.63	29.61

**Table 2:** Comparison of the refined *A* and *B* values

Parameter	(01ν)*	(03ν)	(21ν)*	(23ν)*
*R*_p_	0.012	0.019	0.025	0.020
*A*	0.831	0.842	0.848	0.850
*B*	0.611	0.561	0.721	0.752

## Appendix A

### A1. Correlations of the *s* = 2 model

Given an initial state  , the *n*th state  is calculated by  . To calculate  , the matrix  is diagonalized, *i.e.* expressed as  , where  is a diagonal matrix (Kakinoki & Komura, 1965 ▸). This reduces the problem to calculating  , which is trivial, since the *n*th power of a diagonal matrix is obtained by taking the *n*th power of the diagonal elements. Matrix diagonalization is an *eigenvector* and *eigenvalue* problem. For  of equation (19) we obtain (as one possible solution)

and

where

Given the probabilities of *c*- and *n*-glides according to equations (14) and (15), the initial state of the chain is

and *n*th state is

However, we are not interested in the full state only the sum of the states for which α = 0, 1, that is  . By substitution of equations [(29) and (30)] into equation (33), one obtains:

where

Thus, in general convergence to the equilibrium state  is given by the sum of two power series.

The correlation *c*_Δ*n*_ = 2*P*_Δ*n*_ − 1 is:

Note that if *Q* is negative, (*Q*)^1/2^ and *R* are purely imaginary. However, in that case 1 + *R* and 1 − *R* are complex conjugate, as *c*_1_ and *c*_2_ are. Thus, *c*_Δ*n*_ is the sum of two complex conjugates and therefore real, as expected.

### A2. Diffuse scattering for *h* = 0

Substituting of equation (38) into equation (10, one obtains:

The equivalence of the sums over Δ*n* and the shape functions  and  have for example been derived in Stöger *et al.* (2021 ▸).

For negative *Q*, the terms in equation (40) are complex. Since both terms are complex conjugate, resulting in a positive real intensity, as expected. By using the identities

Equation (40) can also be written using only real terms as

where *C* = *A* + *B* − 2. In the *Q* < 0 case, this can not generally be interpreted in terms of contributions by the *Pca*2_1_ and *Pna*2_1_ polytypes. One exception is *A* = *B*, in which case equation (45) becomes

where *c* = 2*A* − 1 = 2*B* − 1.

### A3. Diffuse scattering for *h* ≠ 0

Substituting of equation (38) into equation (9), one obtains:

Here, *d*′_*c*_ is a family of shape functions that can be derived in analogy to *d*_*c*_:

where  represents the real part.
